# Preparation and Adsorption Properties of Maleic Anhydride-Modified Cellulose Nanofibers

**DOI:** 10.3390/polym17192586

**Published:** 2025-09-24

**Authors:** Jia-Ning Meng, Dan Qiu, Tao Yuan, Ya Li, Huang Huang, Ling-Hui Wang, Ya-Juan Wang, Rui Wang, Chang-Zi Jin

**Affiliations:** 1 School of Petrochemical Engineering, Liaoning Petrochemical University, Fushun 113001, China; 15033020678@163.com (J.-N.M.); wr_wrwr@163.com (R.W.); jin_chz@163.com (C.-Z.J.); 2 School of Biological and Chemical Engineering, NingboTech University, Ningbo 315100, China; 3 School of Materials and Chemical Engineering, Ningbo University of Technology, Ningbo 315211, China; liya@nbut.edu.cn (Y.L.); wanglh@nbut.edu.cn (L.-H.W.); wangyajuan@nbut.edu.cn (Y.-J.W.); 4 Zhejiang Institute of Tianjin University, Ningbo 315201, China; 5 Zhejiang Hanghua New Materials Technology Co., Ltd., Hangzhou 311301, China; hhzq950704@163.com

**Keywords:** maleic anhydride-modified cellulose nanofibers (MA-CNFs), esterification, hydrophobicity, surface area, adsorption

## Abstract

Cellulose nanofibers (CNFs) are highly promising nanocarrier materials, boasting excellent drug adsorption and loading potential due to their tunable hydrophilic/lipophilic interfaces. This study is the first to report the successful synthesis of maleic anhydride-modified CNFs (MA-CNFs) via the esterification of CNFs using a solvent-free molten maleic anhydride (MA) system, and it systematically evaluates MACNFs’ dual adsorption performance for water-soluble and lipophilic drugs. A new characteristic peak at 1723 cm^−1^ in FT-IR confirms the formation of ester bonds, proving the successful grafting of MA onto CNFs. XRD analysis shows that the crystallinity slightly increases from 72.56% to 74.06%, indicating the reaction mainly occurs in the amorphous region. After modification, the material’s hydrophobicity is significantly enhanced (water contact angle: ~63.3° for CNFs vs. ~74.9° for MA-CNFs), and its BET specific surface area rises sharply from 5.03 to 26.29 m^2^/g. These structural advantages collectively enable MA-CNFs to have adsorption capacities for folic acid (FA, water-soluble) and vitamin E acetate (VEA, lipophilic) that are 1.15 and 2.04 times those of CNFs, respectively. The results demonstrate MA-CNFs are high-performance functional materials fabricated via a green method, with good biocompatibility.

## 1. Introduction

The development of lipophilic and hydrophilic drug carriers is a key strategy in contemporary medicine to overcome the limitations of pharmaceuticals and enhance therapeutic outcomes [1,2]. This approach has major implications in various domains, including clinical treatment, industrial advancement, and public health. A common challenge associated with drug carriers is their limited loading capacities [3]. Thus, there is a need to develop carrier materials with enhanced loading capacities for effective drug delivery.

Synthetic and natural polymers are primarily used to fabricate drug carriers. Compared with synthetic polymers, natural polymers offer superior biocompatibility and biodegradability. Consequently, using natural polymers as drug carriers not only facilitates efficient drug delivery but also minimizes the toxic side effects on the organism [4,5]. Therefore, natural polymer materials can be expected to emerge as promising drug carriers owing to their high biocompatibility and substantial loading capacity.

Cellulose, the most abundant natural polymer in the environment, is synthesized globally via photosynthesis at an estimated annual rate exceeding 100 billion tons [6]. Cellulose is derived from a diverse array of sources, including agricultural and forestry by-products—such as wood, cotton, and straw—and is considered to be a sustainable resource with considerable potential for promoting sustainable development [7]. It is characterized by its high biocompatibility, biodegradable nature, and exceptional mechanical properties.

Consequently, the advent of nanotechnology has led to the development of cellulose nanofibers (CNFs), which have considerable application potential in reinforced composites, biosensors, and drug delivery systems. This potential can be attributed to the nanoscale effects of nanomaterials, combined with the inherent advantages and renewability of cellulose [8]. Accordingly, CNFs have emerged as the focal point of research in the field of nanomaterials. The dense hydroxyl groups on the surface of CNFs confer high reactivity and present notable challenges. These hydroxyl groups readily form intermolecular hydrogen bonds, leading to strong interactions among the nanofibers and subsequent agglomeration [9]. The agglomeration of CNFs not only negates the benefits associated with nanoscale dispersion but also compromises the homogeneity and mechanical properties of the material. Moreover, the hydrophilic hydroxyl groups on the surface of CNFs exhibit poor interfacial compatibility with most organic matrices, which has led to weak interfacial bonding between the two phases and seriously limited the application potential of CNFs.

The modification of cellulose nanofibers (CNFs) is of great significance for overcoming limitations such as easy aggregation and poor interfacial compatibility. Currently, the main modification strategies include physical modification [10,11] and chemical modification [12,13]. By improving the dispersibility and interfacial bonding ability of CNFs, these methods have significantly expanded their functional applications. Existing studies have shown that specific chemical modification of cellulose can effectively enhance its adsorption capacity for water-soluble pollutants. For example, Aloulou et al. [14] esterified microcrystalline cellulose with caprylic anhydride and confirmed its excellent adsorption performance for organic molecules in water; Feng et al. [15] prepared carbon nanospheres via hydrothermal carbonization of α-cellulose, which can adsorb trace amounts of diclofenac sodium (DCF) (0.01 mg/mL); Komal et al. [16] modified CNFs synergistically with graphene oxide and oxalic acid, achieving adsorption capacities of 45.04 mg/g and 85.30 mg/g for ciprofloxacin and ofloxacin, respectively; Gopakumar et al. [17] modified CNF-PVDF composite membranes with 2,2-dimethyl-1,3-dioxane-4,6-dione, significantly improving the adsorption capacity for water-soluble substances.

However, most existing modification studies focus on hydrophilic pollutants, while targeted adsorption research on hydrophobic and lipophilic substances remains insufficient, which limits the application of CNFs in broader environmental governance. Notably, maleic anhydride (MA) modification, as an effective biopolymer functionalization strategy, can introduce active functional groups such as carboxyl and ester groups through ring-opening reactions, thereby precisely regulating the surface properties of materials [18]. Although MA modification has shown significant advantages in other cellulose forms—for instance, Zhou et al. [19] used MA to modify mercerized cellulose to enhance its adsorption performance, and Jin et al. [20] improved the compatibility between bamboo fibers and hydrophobic matrices through MA esterification—these studies all face bottlenecks for industrial application, such as complex post-treatment and harsh reaction conditions.

Most importantly, no research has yet systematically explored the modification effect of MA on CNFs at the nanocellulose scale, especially the adsorption behavior and mechanism for lipophilic pollutants. Compared with microscale cellulose or composite substrates, the high specific surface area and accessible hydroxyl density of CNFs provide unique advantages for MA modification, which is expected to break through the balance dilemma between adsorption performance and process feasibility. This study aims to achieve controllable esterification of CNFs using MA, systematically evaluate their adsorption performance for typical lipophilic substances, and clarify the structure-activity relationship and enhancement mechanism during the modification process. This work not only fills the research gap of MA-modified CNFs in the field of hydrophobic adsorption but also provides a new path for the development of high-performance and processable cellulose-based adsorption materials.

In this study, cellulose nanofibers (CNFs) were used as the substrate and chemically modified with maleic anhydride (MA), addressing the critical gap that no previous research has reported the use of MA-modified CNFs to achieve efficient simultaneous adsorption of hydrophilic and lipophilic drugs. This study systematically carried out the following work: First, multiple characterization techniques were employed to analyze the changes in the microstructure of MA-CNFs and the modification effect of functional groups. Second, folic acid (FA), a water-soluble drug, and vitamin E acetate (VEA), a lipid-soluble drug, were selected as model compounds, and the amphiphilic drug adsorption performance of MA-CNFs was evaluated through static adsorption experiments. Finally, the contribution of active functional groups introduced by MA to the drug adsorption mechanism was explored in depth. he results showed that MA modification significantly improved the adsorption efficiency of CNFs for both FA and VEA. This study not only reveals the structural evolution law and drug adsorption mechanism of MA-CNFs but also provides a theoretical basis and technical support for the application of CNFs in drug delivery systems, which is expected to expand the development prospects of biomass nanomaterials in the field of functional materials.

## 2. Materials and Methods

### 2.1. Materials

The CNFs used in this study had a solid content of 3% and were procured from Hanghua New Materials Technology Co., Ltd. (Hangzhou, China). Analytical-grade MA was procured from Aladdin Reagent Co., Ltd. (Shanghai, China); analytical-grade dichloromethane was procured from Sinopharm Chemical Reagents Co., Ltd. (Shanghai, China); analytical-grade anhydrous ethanol was procured from Shanghai Titan Technology Co., Ltd. (Shanghai, China); chromatographically pure deuterated DMSO (DMSO-D6) was procured from Shanghai Yuanye Biotechnology Co., Ltd. (Shanghai, China); analytical-grade FA was procured from Shanghai Maclean Biochemical Technology Co., Ltd. (Shanghai, China); analytical-grade anhydrous sodium carbonate was procured from Sinopharm Chemical Reagents Co., Ltd.; analytical-grade potassium dihydrogen phosphate was procured from Sinopharm Chemical Reagent Co., Ltd.; analytical-grade potassium bromide was procured from Shanghai Maclean Biochemical Technology Co., Ltd.; and food-grade Vitamin E acetate was procured from Jiangsu Meique Biotechnology Co., Ltd. (Zhenjiang, China).

### 2.2. Esterification Modification of CNF

**Figure 1:**
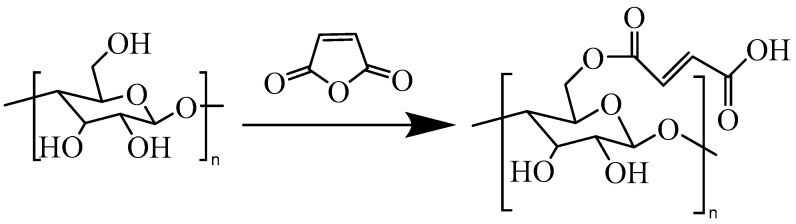
Reaction of maleic anhydride (MA) with the cellulose nanofibers (CNFs).

Figure 1 shows the reaction equation of maleic anhydride and CNF. Fifteen grams of maleic anhydride was taken and added into a 50 mL round-bottomed flask, which was then heated in a 90 °C water bath to melt the maleic anhydride (this temperature and the corresponding reaction time were determined based on pre-experiments and literature optimization [21], which can ensure that maleic anhydride stays in a molten state, promote the effective esterification reaction between maleic anhydride and the hydroxyl groups on the cellulose surface, and avoid its excessive decomposition at the same time). Subsequently, 1 g of freeze-dried cellulose nanofibers (CNF) was added to the flask, and a continuous reaction was carried out for 3 h under mechanical stirring at 500 r/min. After the reaction was completed, hot filtration was performed to remove unreacted maleic anhydride, and the product was washed repeatedly with deionized water for 3 times. To further remove residual maleic anhydride, the product was subjected to suction filtration and washing three times using 3 portions of 20 mL dichloromethane (Analytical Reagent, ≥99.5%). Finally, the purified product was freeze-dried for 48 h to obtain maleic anhydride-modified cellulose nanofibers (MA-CNF).

### 2.3. Characterization

Proton nuclear magnetic resonance (^1^H NMR, AVANCEIIIHD500MHz, USA) spectroscopy was used to determine the presence of MA in the filtrate. Then, 0.5 mL of the filtrate was dissolved in 3 mL of deuterated dimethyl sulfoxide (DMSO-D_6_, chromatographic grade, ≥99.5%) for analysis. Fourier transform infrared spectroscopy (FTIR, Nicolet iS10, USA) was used to identify the characteristic functional groups of cellulose via the potassium bromide (KBr) pellet method. The sample was ground with dry KBr at a mass ratio of 1:100, and the mixture was pressed into a pellet. Spectra were recorded over a wavenumber range of 4000–500 cm^−1^ with a resolution of 4 cm^−1^ and 64 scans. The surface morphologies of the samples were then examined using scanning electron microscopy (SEM; Phenom Pro, USA). The samples were affixed to the sample stage using a conductive adhesive and subjected to gold sputtering in a vacuum at an acceleration voltage of 15 kV. The crystalline properties of the materials were analyzed using X-ray diffraction (XRD, D8 Advance, DE) with a tube voltage of 40 kV and a current of 27 mA. The scanning rate was set at 5°/min, covering a 2θ range of 10–50°.

The contact angle of the material was measured using a water contact angle device (WCA, OCA15EC, DE). The CNF (0.06 g) and MA-CNF powders were weighed and dispersed in 60 mL of deionized water. This dispersion was ultrasonicated at 80% power using an ultrasonic cell disruptor for 1 h, followed by filtration through a polycarbonate membrane, which was sectioned at three distinct locations for measurement purposes. The N2 physical adsorption was performed on samples. The samples underwent nitrogen adsorption at 77 K and vacuum degassing at 120 °C for 6 h. The specific surface area were evaluated using the Brunauer–Emmett–Teller (BET) method.

### 2.4. Determination of Degree of Substitution

Optimizations were implemented in accordance with the methodology outlined in the literature [22]. In the literature, the color change in an indicator was used as the end point of the reaction. This operation resulted in relatively large reaction errors, and the color was difficult to control. After optimization, a pH meter was adopted, which is more accurate. A dry MA-CNF sample (1.00 g) was placed in a 250 mL conical flask. Subsequently, 10 mL of 75% ethanol and 10 mL of a 0.5 mol/L NaOH solution were added, maintaining the mixture at 30 °C under constant stirring for 30 min. Subsequently, the excess base present in the conical flask was titrated using a 0.5 mol/L hydrochloric acid solution. The volume of hydrochloric acid was documented when the titration endpoint was reached and denoted as *V*_1_. As a blank control, an equivalent mass of CNFs was used to record the volume of hydrochloric acid required to reach the endpoint, denoted as *V*_0_. The following formula can be used to calculate the DS of the MA-ST: All titration experiments were independently repeated three times, and the degree of substitution results were expressed as mean ± standard deviation (SD).

$$C_{MA}/\%=98\times c\times (V_{0}-V_{1})/(1000\times 2m)\times 100$$

(1) $$DS=162\times C_{MA}/[98\times 100\times (1-C_{MA})]$$

where *C_MA_* denotes the MA content, *m* denotes the mass of the MA-CNF sample (g), *c* denotes the hydrochloric acid concentration (mol/L), *V*_0_ denotes the volume of hydrochloric acid consumed during the blank titration process (mL), and *V*_1_ denotes the volume of hydrochloric acid consumed during the sample titration process (mL).

### 2.5. Determination of Adsorption Performance

The method for testing the performance of the FA adsorption performance was optimized based on the established literature [23]. The FA (10.0 mg) was dissolved in a 0.1 mol/L sodium carbonate solution, and the volume was then adjusted to the mark using a 10 mL amber volumetric flask. Next, 0.05, 0.10, 0.20, 0.40, and 0.60 mL of this solution was transferred into separate 50 mL amber volumetric flasks and each flask was filled with a metaphosphoric acid buffer. The concentration range of the solution is 1 μg/mL, 2 μg/mL, 4 μg/mL, 8 μg/mL, and 12 μg/mL. The absorbance was measured at 281 nm using UV spectrophotometry, yielding a standard curve described by the equation: Y = 0.06654*X* − 0.02444, where Y denotes the absorbance and *X* denotes the FA concentration (R^2^ = 0.99996).

Subsequently, another 10.0 mg of FA was weighed and dissolved in a 0.1 mol/L sodium carbonate solution and then diluted in a 100 mL amber volumetric flask. Next, 10.00 mL was transferred to separate containers, containing 10.0 mg of MA-CNFs and CNFs, the concentration of the solution is 10 μg/mL. The mixtures were incubated in the dark for 24 h to facilitate adsorption. Solid–liquid separation was achieved by centrifugation at 8000 rpm for 5 min. After centrifugation, 5 mL of the supernatant was accurately pipetted and diluted to 50 mL in an amber volumetric flask. The measurement was repeated three times, and the calculation was performed according to the following formula. The adsorption capacity results were expressed as mean ± standard deviation (SD).

(2) $$q=\frac{\left(C_{0}-C\right)v}{m}$$

where q denotes the adsorption capacity (mg/g), *C*_0_ denotes the initial concentration of the solution before adsorption (0.01 mg/mL), *C* denotes the equilibrium concentration of the solution after adsorption (mg/mL), *v* denotes a constant volume (mL), and *m* denotes the mass of the CNFs and MA-CNFs (g).

The performance test method for adsorbing lipid-soluble drugs was as follows: 100 mg of VEA was dissolved in ethanol, made up to 50 mL, before 0.50, 1.00, 1.50, 2.00, and 10 mL of this solution was transferred into separate 50 mL amber volumetric flasks and filled with ethanol. The concentration range of the solution is 0.1 mg/mL, 0.2 mg/mL, 0.3 mg/mL, 0.4 mg/mL, and 0.5 mg/mL The absorbance was measured at 292 nm using UV spectrophotometry, and the standard curve, Y = 1.8816*X* + 0.00628 was plotted, where Y denotes the absorbance and *X* denotes the mass concentration of VE (mg/mL) (R^2^ = 0.99995). Next, 100 mg of the VE was dissolved in anhydrous ethanol. The solution was transferred to a 50 mL volumetric flask and filled with ethanol, the concentration being 2.00 mg/mL. Ten mL of the solution was transferred to 20.0 mg of MA-CNF powder and allowed to stand in the dark for 24 h to facilitate adsorption. Solid–liquid separation was achieved by centrifugation at 8000 rpm for 5 min. After centrifugation, 1 mL of the supernatant was accurately pipetted and diluted to 10 mL in an amber volumetric flask. The absorbance of the solution was measured at a wavelength of 292 nm, and the same mass of CNF powder was used as a blank control test for content determination. The measurement was repeated three times, and the calculation was performed according to the following formula. The adsorption capacity results were expressed as mean ± standard deviation (SD).

(3) $$q=\frac{\left(C_{0}-C\right)v}{m}$$

where q denotes the absorbance (mg/g), *C*_0_ denotes the initial concentration of the solution before adsorption (0.20 mg/mL), *C* denotes the equilibrium concentration of the solution after adsorption (mg/mL), *v* denotes a constant volume (ml), and *m* denotes the mass of the CNFs and MA-CNFs (g).

## 3. Results and Discussion

### 3.1. Chemical Analyses

Because MA undergoes esterification in the molten state, unreacted MA is easily adsorbed by CNFs, which can interfere with subsequent structural characterization. Therefore, the experimental process was optimized in this study, and the washing effect of MA was reflected in the ^1^H NMR spectrum of dichloromethane.

**Figure 2:**
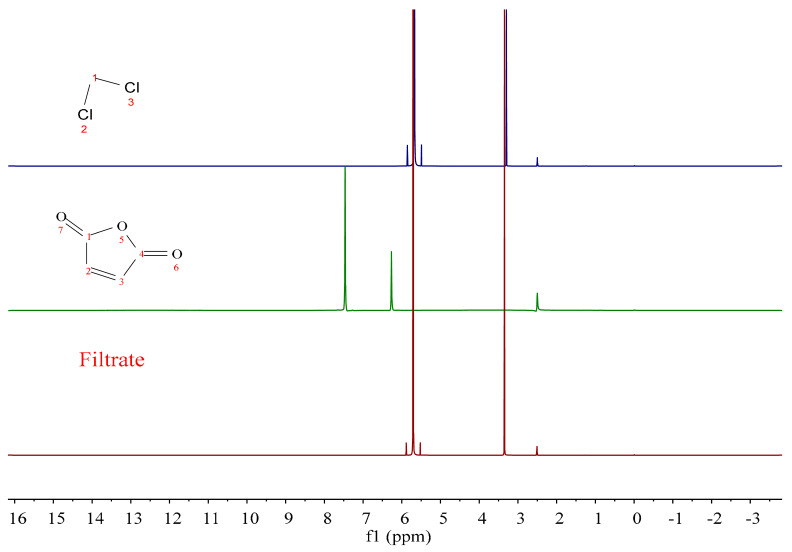
H^1^ NMR spectra of the dichloromethane, MA and washing solution.

Figure 2 shows the ^1^H NMR spectra of dichloromethane, MA, and the washing solution. Table 1 shows the chemical shift values of the ^1^H NMR spectra of dichloromethane and MA. It is evident that the main characteristic peak of dichloromethane was at δ = 5.68, which was the proton signal peak on -CH_2_; the DMSO-D6 signal peak was at δ = 2.50–2.52, and the MA characteristic peak was at δ = 7.45. By comparison, it was evident that the characteristic peak of the washing solution after multiple washes was consistent with that of dichloromethane, indicating that there was no residual MA in the MA-CNF sample. Thus, washing with dichloromethane proved to be an effective method for eliminating the interference caused by unreacted MA.

**Figure 3:**
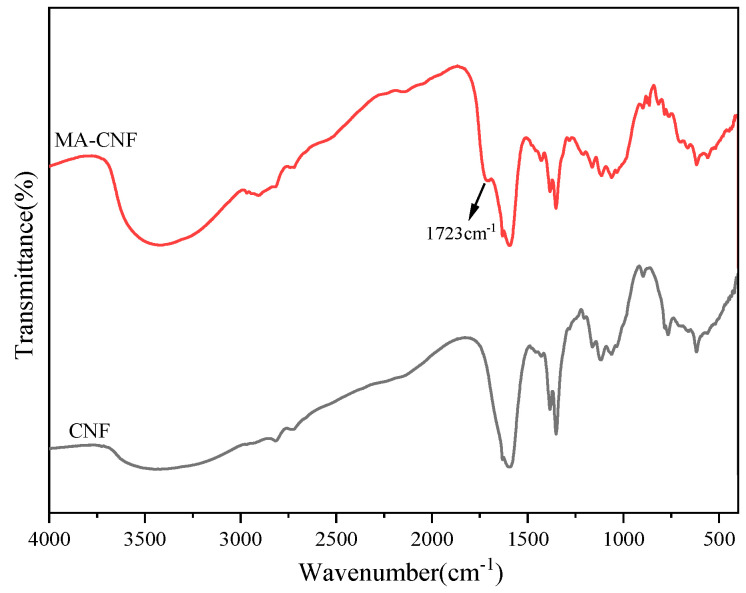
FTIR spectra of CNF versus MA-CNF.

The degree of substitution of the MA-CNFs was 0.108 ± 0.001, indicating successful esterification of the CNFs. As illustrated by the infrared spectral data shown in Figure 3, the CNFs exhibited a stretching vibration peak of cellulose -OH at 3428 cm^−1^ and an asymmetric stretching vibration peak of C-H at 2750 cm^−1^. The absorption peak at 1593 cm^−1^ corresponded to the bending vibration of O-H, whereas the absorption peak at 1350 cm^−1^ could be attributed to the asymmetric stretching vibration of C-O-C.

After the esterification modification of CNFs with maleic anhydride (MA), the stretching vibration peak of -OH at 3428 cm^−1^ is significantly enhanced. This is because side-chain carboxylic acid groups (-COOH) are introduced onto the cellulose chains during esterification. The carboxylic acid groups themselves also contain hydroxyl groups (-OH), whose stretching vibration peaks also appear around 3500 cm^−1^. Due to the strong hydrogen-bonded dimer effect, this broad peak overlaps severely with the hydroxyl peak of the CNFs themselves, thus leading to the enhancement of the vibration peak. Concurrently, a new characteristic signal emerged near 1723 cm^−1^, attributed to the stretching vibration of the C-O bond of the esterification group. The characteristic peak of the C=O group of MA could be observed within the 1700–1750 cm^−1^ range. This was consistent with the position of the characteristic peak in the study of MA esterification of cellulose by Fridrihsone [24], indicating that MA successfully esterified the CNFs and connected them to the hydroxyl group of CNFs in the form of ester bonds.

**Figure 4:**
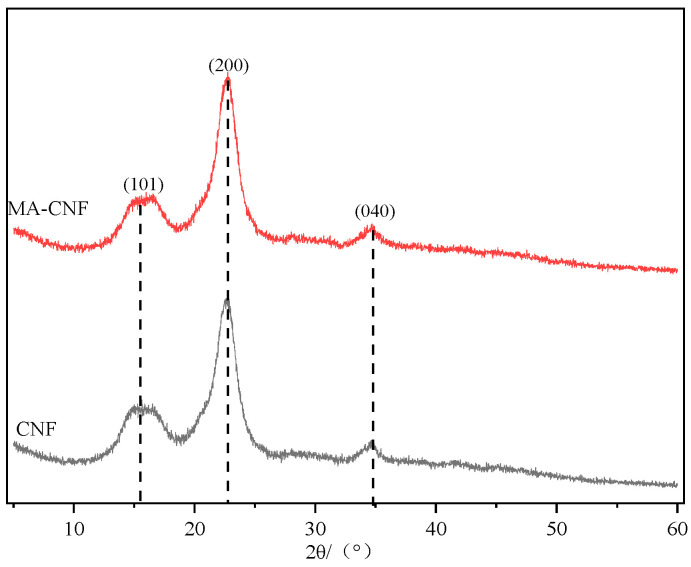
XRD patterns of CNF and MA-CNF.

The XRD pattern presented in Figure 4 shows the characteristic peaks of the original CNFs at 2θ angles of 15.8°, 22.8° and 34.7°, corresponded to (101), (200) and (040) planes of cellulose, respectively. Although the intensities varied, the positions of these diffraction peaks in the MA-CNFs remained unchanged, suggesting that the crystal structure of CNFs was preserved after MA esterification.

Using the method proposed by Segal [25], the crystallinity of the CNFs was determined to be 72.56%, whereas that of the MA-CNFs increased to 74.06%. A similar phenomenon was reported in the previous literature [26], where the crystallinity index of cellulose pulp increased from 72.5% to 79.3% after maleic anhydride (MA) esterification. This indicates that more amorphous cellulose regions were hydrolyzed during the esterification process. The molecular chains in these regions are loosely and disorderly arranged, with high reactivity; their structure changes after esterification, leading to an increase in the relative proportion of crystalline regions and thus a slight enhancement in the overall crystallinity. This further confirms that mild esterification conditions can effectively retain the crystal structure of cellulose nanofibers (CNFs) while achieving selective modification of the amorphous regions.

### 3.2. Characteristics of Structural Properties for MA-CNF

**Figure 5:**
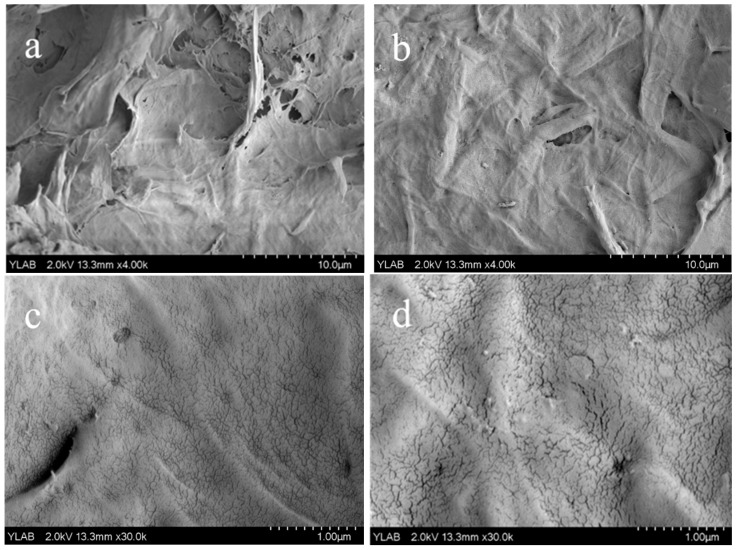
SEM images of cellulose nanofibers CNF (**a**,**c**) and maleic anhydride-modified cellulose nanofibers MA-CNF (**b**,**d**).

Figure 5a illustrates that the CNFs tended to agglomerate following freeze-drying, forming bundles owing to hydrogen bonding, which resulted in a fibrous surface structure. This observation was consistent with previously reported electron microscopy results [27]. The morphology changed, rendering the CNF surface smoother after modification. As depicted in Figure 5c, the surface of the CNFs exhibited dense small pores, with the number of these pores increasing after the esterification process (Figure 5d). This increase could be attributed primarily to the disruption of the original hydrogen bond network by the esterification process, leading to alterations in the interstitial structure of the CNFs and a notable enhancement of the porous structure.

**Figure 6:**
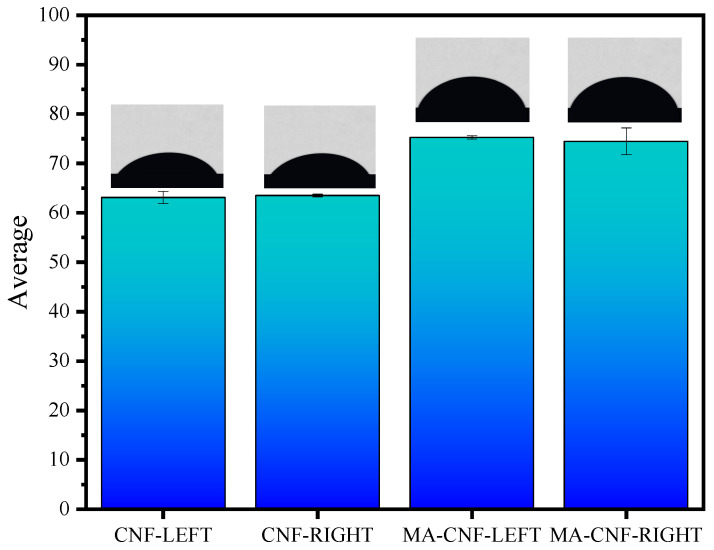
Bar chart of water contact Angle between CNF and MA-CNF.

Three different positions on the CNF and MA-CNF membranes were cut and measured using a contact angle meter, the recorded data of which are shown in Figure 6.

As is evident from the WCA data, the WCAs of the MA-CNFs (75.26–74.46°) were higher than those of the CNFs (63.10–63.50°), and both the left and right WCAs improved after esterification. This was similar to a study in which nanocrystalline cellulose was modified by grafting with long-chain fatty acids to increase its WCA and improve its hydrophobicity, indicating that CNFs modified by esterification exhibited a certain hydrophobic capacity [28]. The increase in contact angle is attributed to the introduction of long alkyl chains (derived from maleic anhydride) and the change in hydrogen bond network, which collectively lead to a decrease in surface energy. However, the enhanced hydrophobicity may exert adverse effects on certain applications. For instance, in aqueous drug delivery systems, excessively high hydrophobicity can lead to difficulties in the dispersion of nanofibers, which in turn impairs drug loading efficiency and release behavior. Therefore, when designing drug carriers, it is necessary to balance hydrophobic modification and dispersion stability.

**Figure 7:**
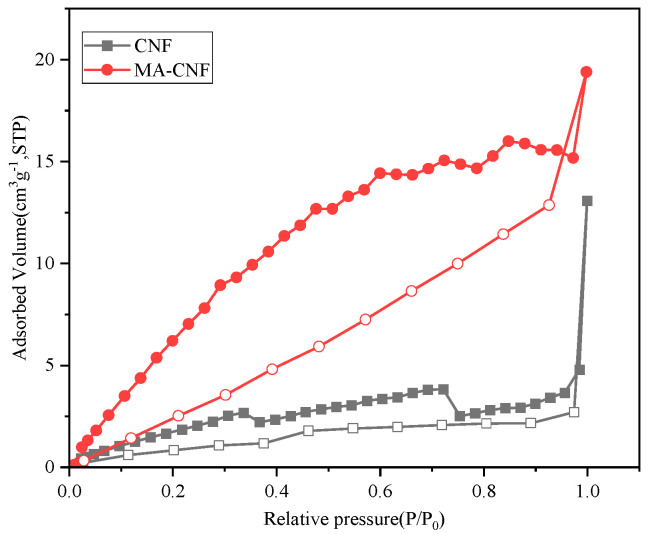
Isothermal curves of adsorption and desorption of CNF and MA-CNF.

The porous structures of the CNFs and MA-CNFs were further analyzed using N_2_ absorption/desorption isothermal curves (Figure 7 and Table 2). It is evident that both the CNFs and MA-CNFs exhibited type II adsorption/desorption isotherms with type H3 hysteresis loops. The H3-type hysteresis loop indicated the presence of cylindrical pores in the sample, which were also evident in the CNF/chitin nanofiber sheets [29]. The pores originated primarily from the CNFs and were hypothesized to be fiber pores formed after the freeze-drying process.

**Figure 8:**
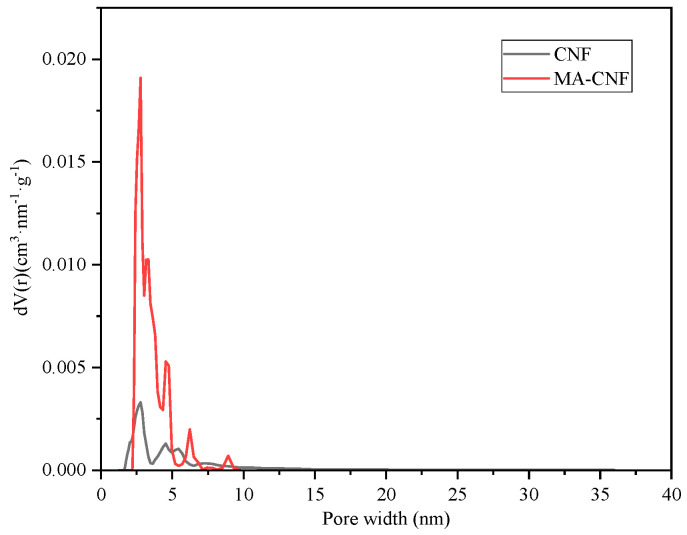
NLDFT (Non-local Density Functional Theory) pore size distribution map of CNF and MA-CNF.

As illustrated in Figure 8, the pore size distribution of the CNFs fell predominantly within the mesoporous and microporous ranges, with micropores of approximately 1.6 nm in size and mesopores ranging from 2.1 to 3.1 nm in size. By contrast, the pore size distribution of the MA-CNFs was chiefly within the mesoporous range, spanning 2.3–4.9 nm, and lacked a microporous structure. This alteration could be attributed to the increase in the effective pore size resulting from esterification. The significant increase in pore size exerts a crucial impact on drug loading behavior, particularly for drugs with different solubility properties. For water-soluble drugs, the expanded mesopore range of MA-CNFs provides a larger specific surface area and more diffusion pathways, which is conducive to enhancing drug loading capacity and promoting drug release in aqueous environments. However, since esterification modification simultaneously enhances the hydrophobicity of the material, it may partially weaken its adsorption affinity for hydrophilic drugs. For fat-soluble drugs, the increased pore size and hydrophobic modification exhibit a synergistic effect: wider mesopores facilitate the accommodation of hydrophobic drug molecules, while the improved surface hydrophobicity of the material also enhances its compatibility and adsorption with fat-soluble drugs. As a result, the drug loading efficiency and drug-loading stability are significantly improved.

As indicated in Table 2, the BET specific surface area of the CNFs was 5.03 m^2^/g, which was relatively low, possibly owing to agglomeration after the freeze-drying process. The BET surface area of the CNFs increased from 5.03 to 26.29 m^2^/g after esterification, representing a 4.23-fold increase, whereas the pore volume expanded by a factor of 2.14. This enhancement could be attributed to the disruption of the hydrogen bond structure within the CNFs owing to esterification, leading to an enlarged pore volume and an increased number of pores. These findings demonstrate that esterification had a considerable influence on the BET surface area and pore volume of the CNFs.

### 3.3. Characterization and Adsorption Performance Tests

**Figure 9:**
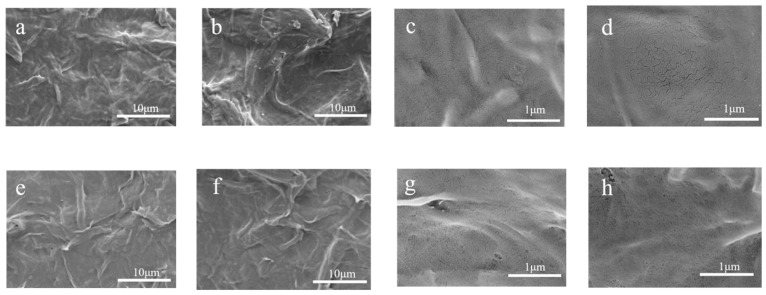
SEM of CNF-FA (**a**,**c**), MA-CNF-FA (**b**,**d**) and CNF-VEA (**e**,**g**), MA-CNF-VEA (**f**,**h**).

Following the adsorption of FA and VE acetate by the CNF, the fiber structure remained unchanged, as illustrated in Figure 9a,b,e,f, when compared to Figure 5a,c. (Note: The magnification and scale bar of the electron micrograph in this figure are consistent with those in Figure 5a–d) However, upon magnification, a reduction in the pore structure of the fiber was observed, with a more pronounced decrease evident in MA-CNF, as shown in Figure 9c,d,g,h, compared to Figure 5b,d. This observation aligns with previous findings of previous research [30] that reported similar electron microscopy morphological changes in CMC-based microspheres upon VC adsorption.

**Figure 10:**
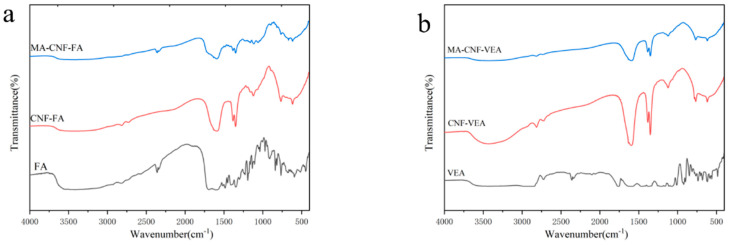
FTIR spectra of CNF and MA-CNF adsorbing folic acid (**a**) and VE acetate (**b**).

As is evident in Figure 10a, the infrared characteristic peaks of FA were located at 1696, 1606, and 1485 cm^−1^, corresponding to the vibration absorption peaks of the carboxyl group, amino group on the pteridine ring, C-C and C-N [31]. The cellulose structures of the CNFs and MA-CNFs remained unchanged after the adsorption of FA, and the contrast raw materials exhibited enhanced peaks to varying degrees. The characteristic peak of MA-CNF adsorbed FA at 1709 cm^−1^ was higher than that of the CNFs, indicating that the MA-CNFs exhibited a stronger adsorption performance for water-soluble FA.

The infrared characteristic peaks of VEA (Figure 10b) were at 2830 cm^−1^ (C-H stretching), 1763 cm^−1^ (C=O stretching), 1480 cm^−1^ (CH_2_ bending), and 1057 cm^−1^ (C=O stretching) [32]. The cellulose structures of the CNFs and MA-CNFs adsorbed with VEA remained unchanged, although a characteristic peak appeared near 1125 cm^−1^, owing to the C=O stretching peak of VEA, which indicated that both the CNFs and MA-CNFs had certain adsorption properties for lipophilic VEA.

**Figure 11:**
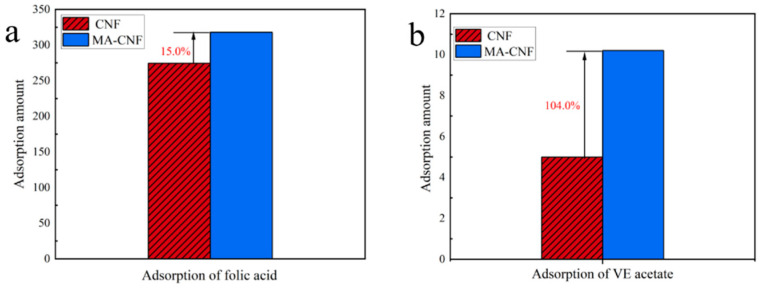
Graphical analysis of CNF and MA-CNF adsorption capacities for folic acid (**a**) and VE acetate (**b**).

Based on the adsorption capacity, as expressed in Equation (2), the FA adsorption capacity of the CNFs was determined to be 274.5 ± 4.00 mg/g. As illustrated in Figure 11a, the MA-CNFs exhibited a FA adsorption capacity of 318.0 ± 5.25 mg/g, representing an increase of 1.15 times compared to that of the CNFs. Using Equation (3), the VEA adsorption capacity of the CNFs was determined to be 5.00 ± 0.13 mg/g. The MA-CNFs exhibited a VEA adsorption capacity of 10.20 ± 0.68 mg/g, which was 2.04 times higher than that of the CNFs (Figure 11b). Currently, there are relatively few reported studies on the adsorption of vitamin E acetate (VEA) in the literature. Marnani et al. [33] investigated the adsorption of folic acid (FA) in diphenylalanine peptide nanopores, but did not specify the relevant adsorption capacity, which may be due to poor adsorption performance.

These findings indicate that the modified CNFs exhibited superior adsorption capacity for the lipophilic drug VEA compared to the water-soluble drug FA. This enhancement effect mainly stems from two key factors: first, the esterification process significantly improves the hydrophobicity of the material. Through the replacement of hydroxyl groups (-OH) with ester groups (-COO-), the surface property of the material is transformed from hydrophilic to hydrophobic, which enhances its affinity with low-polarity drugs; second, the nanofiber structure provides a large specific surface area, offering abundant sites for drug adsorption. From the perspective of molecular mechanism, the adsorption between MA-CNFs and folic acid (FA) may be achieved through specific interactions such as hydrogen bonds formed between carboxyl groups and amino groups, as well as π-π stacking between aromatic rings. In contrast, the binding between MA-CNFs and vitamin E acetate (VEA) mainly relies on hydrophobic interactions.

Although MA-CNFs exhibit promising drug-loading potential, their practical application still faces several challenges: the easy hydrolysis of ester bonds in acidic/alkaline environments may affect the long-term stability of the material; the strong interaction between drugs and the carrier may lead to desorption difficulties, restricting its reusability; the toxicological behavior of residual reagents during the modification process and nano-scale fibers still requires systematic evaluation to ensure biocompatibility and environmental safety. Future research should focus on improving the chemical stability of the material, developing mild and efficient desorption methods, and conducting comprehensive toxicological studies.

## 4. Conclusions

In this study, we successfully prepared MA-CNFs by esterification modification of CNFs with MA in the molten state. The reaction system capitalized on the low melting point of MA, facilitating esterification of the hydroxyl groups on the surface of the CNFs with the carboxyl groups of MA in a molten state devoid of organic solvents. This approach enabled esterification under mild conditions. Compared to traditional methods, the proposed technique not only simplified the reaction process and diminished the environmental impact associated with organic solvents, but also allowed for the efficient recovery and reuse of unreacted MA monomers through a straightforward procedure. Following a comprehensive analysis of the modified MA-CNFs using SEM, XRD, and BET techniques, several key findings were obtained. The SEM images revealed that the surface of the modified MA-CNFs exhibited a pronounced fold and pore structure, which provided an increased number of active sites for drug adsorption compared to the unmodified CNFs. XRD pattern analysis demonstrated that the esterification process did not compromise the crystalline structure of the CNFs but instead favored the amorphous cellulose regions, resulting in an increase in crystallinity from 72.56% to 74.06%. Moreover, BET analysis indicated that the specific surface area of the modified CNFs increased by a factor of 4.23. Additionally, the WCA of the MA-CNFs (75.26–74.46°) were considerably higher than those of the CNFs (63.10–63.50°), suggesting a substantial enhancement in its hydrophobic properties. In the drug adsorption performance study, the water-soluble drug FA and lipid-soluble drug VEA were used as models. The findings indicated that the adsorption capacity of the MA-CNFs was 1.15 and 2.04 times greater than that of the CNFs for FA and VEA, respectively. The esterified modified CNFs demonstrated dual adsorption capabilities. This novel MA-CNF nanomaterial, characterized by high drug-loading capacity and superior hydrophobicity, has enormous potential for biomedical applications. Although this material exhibits high drug-loading capacity and hydrophobic advantages, its practical application still faces certain limitations, including the lack of evaluations on in vitro and in vivo drug release behavior as well as biocompatibility, the unproven feasibility of large-scale preparation, and the unknown environmental fate. Future research can focus on aspects such as the evaluation of in vitro/in vivo drug release performance, drug-loading studies on more types of drugs, process scale-up tests, and analysis of environmental degradation and toxicological behavior.

## Figures and Tables

**Table 1:** Chemical shift values of ^1^H NMR spectra of dichloromethane and MA.

Substance	Chemical Shift Range (ppm)	Peak Assignment
* 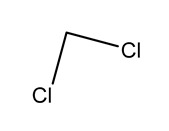 *	~2.49–2.51	DMSO-d6
	~3.70–3.72	HDO
	~5.3–5.5	-CH_2_
* 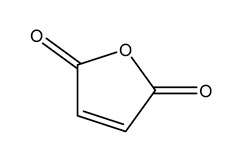 *	~2.50	DMSO-d6
	~3.30–3.50 ~6.2–6.5 ~7.45–7.66	HDO -CH=CH- Hydrogens of unsaturated structures in impurities (peak of hydrolysis product maleic acid)

**Table 2:** Pore structure parameters of CNF and MA-CNF.

Sample	S_BET_ (m^2^·g^−1^)	Pore Volume (cm^3^·g^−1^)
**CNF**	5.03	0.007
**MA-CNF**	26.29	0.022

## Data Availability

The original contributions presented in this study are included in the article. Further inquiries can be directed to the corresponding authors.

## Abbreviations

The following abbreviations are used in this manuscript:

CNFs	Cellulose nanofibers
MA-CNFs	Maleic anhydride-modified cellulose nanofibers
MA	Maleic anhydride
FA	Folic acid
VEA	Vitamin E acetate
FTIR	Fourier transform infrared spectroscopy
^1^H NMR	Proton nuclear magnetic resonance spectroscopy
XRD	X-ray diffraction
WCA	Water contact angle
SEM	Scanning electron microscopy
BET	Brunauer–Emmett–Teller (BET) method.
